# “If I use family planning, I may have trouble getting pregnant next time I want to”: A multicountry survey-based exploration of perceived contraceptive-induced fertility impairment and its relationship to contraceptive behaviors^☆^^☆☆^

**DOI:** 10.1016/j.conx.2023.100093

**Published:** 2023-03-23

**Authors:** Suzanne O. Bell, Celia Karp, Caroline Moreau, PMA Principal Investigators Group, Alison Gemmill

**Affiliations:** a Department of Population, Family and Reproductive Health, Bloomberg School of Public Health, Johns Hopkins University, Baltimore, MD, USA; b Soins et Santé Primaire, CESP Centre for Research in Epidemiology and Population Health U1018, Inserm, Villejuif, France

**Keywords:** Contraceptive use, Low-resource settings, Perceived fertility impairment, Sub-Saharan Africa, Survey research

## Abstract

**Objectives:**

We aim to assess women’s perceptions regarding contraceptive effects on fertility across a diversity of settings in sub-Saharan Africa and how they vary by women’s characteristics. We also aim to examine how such beliefs relate to women’s contraceptive practices and intentions.

**Study design:**

This study uses cross-sectional survey data among women aged 15 to 49 in nine sub-Saharan African geographies from the Performance Monitoring for Action project. Our main measure of interest assessed women’s perceptions of contraceptive-induced fertility impairment. We examined factors related to this belief and explored the association between perceptions of contraceptive-induced fertility impairment and use of medicalized contraception (intrauterine device, implant, injectable, pills, emergency contraception) and intention to use contraception (among nonusers).

**Results:**

Between 20% and 40% of women across study sites agreed or strongly agreed that contraception would lead to later difficulties becoming pregnant. Women at risk of an unintended pregnancy who believed contraception could cause fertility impairment had reduced odds of using medicalized contraception in five sites; aORs ranged from 0.07 to 0.62. Likewise, contraceptive nonusers who wanted a/another child and perceived contraception could cause fertility impairment were less likely to intend to use contraception in seven sites, with aORs between 0.34 and 0.66.

**Conclusions:**

Our multicountry study findings indicate women’s perception of contraceptive-induced fertility impairment is common across diverse sub-Saharan African settings, likely acting as a deterrent to using medicalized contraceptive methods.

****Implications**:**

Findings from this study can help improve reproductive health programs by addressing concerns about contraception to help women achieve their reproductive goals.

## 1 Introduction

Deciding if and when to have children increasingly involves the use of medicalized interventions and treatment to prevent or enhance the chance of pregnancy. While effective in preventing pregnancy, the use of hormonal contraceptive methods and intrauterine devices (IUDs) that interfere with natural reproductive functions may raise public concerns over their potential impact on future reproductive capacity [1], [2], [3], [4], [5], [6]. Evidence indicates no difference in time-to-pregnancy following contraceptive discontinuation for most methods; however, some research suggests short-term reductions in fecundity for users of some hormonal methods—most consistently injectable contraceptive users—compared to users of barrier or traditional methods [7], [8].

Concerns about impaired fertility are common reasons for contraceptive nonuse in sub-Saharan Africa (SSA) [1], [2], [3], [4], [5], [6], where childbearing is central to women’s social status. We use the term “impaired fertility” to refer to anticipated, subjective, or objective delays becoming pregnant, ranging from delayed return to fecundity following hormonal contraceptive use to medically defined infertility (i.e., the inability to conceive after 12 months of unprotected sex). Women who experience impaired fertility are often blamed and suffer myriad health and social consequences as a result [9], [10], [11], [12], [13].

Improved understanding of women’s beliefs about how contraception may affect their future fertility [14], [15] is important in guiding patient-centered care that addresses women’s concerns. This knowledge is particularly relevant for programs in SSA, where use of contraception among those wishing to avoid pregnancy is often low, unintended pregnancies are common, and legal restrictions limit access to safe abortion, thereby hindering women’s ability to achieve their reproductive goals [16], [17], [18].

Existing research on the link between individual concerns about perceptions of contraceptive-induced fertility impairment and contraceptive practices has several limitations. Two systematic reviews highlighting the importance of infertility fears on contraceptive decisions rest solely on qualitative research [2], [5], limiting the generalizability of findings. Available quantitative evidence provides an incomplete picture by focusing on specific subpopulations or contraceptive methods, which hampers our understanding of how widely shared this sentiment is at the population level. For example, a systematic review of quantitative research mostly focuses on infertility concerns related to the IUD and draws largely on nonrepresentative samples of the population or providers from facilities, existing users, or specific subnational geographies [3]. There is also little quantitative research on these issues in SSA, where conception delays may have particularly negative social repercussions.

To address this gap, we aim to assess women’s perceptions about contraceptive effects on fertility across a diversity of settings in SSA and how they vary by women’s characteristics. We also aim to examine how such beliefs relate to women’s contraceptive practices and intentions.

## 2 Material and methods

### 2.1 Data and study settings

This study uses cross-sectional data from the Performance Monitoring for Action (PMA) project, which conducts population-based surveys to track sexual and reproductive indicators among women aged 15 to 49. We focus on nine SSA geographies, including Burkina Faso, Cote d′Ivoire, two provinces from the Democratic Republic of the Congo (Kinshasa and Kongo Central), Kenya, Niger, two Nigerian States (Kano and Lagos), and Uganda; we excluded Rajasthan, India, which is outside SSA, and Ethiopia, which involves a different sampling design and survey. These sites reflect a diversity of reproductive behaviors and norms across SSA, with modern contraceptive prevalence rates ranging from 11.7% in Nigeria to 42.5% in Kenya and total fertility rates ranging from 3.4 in Kenya to 6.8 in Niger [19], [20].

PMA uses a stratified multistage cluster sampling design with probability proportional to size sampling of clusters to produce nationally and/or subnationally representative samples of households and reproductive-aged women. Interviewers map and list all households in selected clusters, which are geographic administrative units comprised of approximately 200 households. A random sample of 35 households is then selected from each cluster sampling frame, and all women aged 15 to 49 identified in selected households are invited to participate in the survey. Samples sizes were calculated based on the sample needed to estimate modern contraceptive prevalence within a three-percentage point margin of error. Sampling methodology is described in greater detail elsewhere [21]. Data used for the analysis were collected face-to-face by local, trained female interviewers between December 2019 and April 2021. Women provided their informed consent to participate in accordance with local ethical committee approvals. Our study was a secondary analysis of these existing data and was thus exempt from additional ethical review and approval. Surveys lasted approximately 45 minutes and included questions about women’s socioeconomic characteristics, reproductive histories, and knowledge of and experience using contraception. Final samples of women ranged from 1112 in Kano, Nigeria to 9478 in Kenya nationally, with response rates all above 95%.

### 2.2 Measurement

Our main measure of interest assessed women’s perceptions of contraceptive-induced fertility impairment. Interviewers asked all participants the extent to which they agreed or disagreed with the statement, “If I use family planning, I may have trouble getting pregnant next time I want to,” using 5-category Likert scale response options. The item was generated from a multicountry mixed-method study on women’s and girls’ sexual and reproductive health empowerment in SSA, which included qualitative in-depth interviews and focus group discussions with 320 women and men from Ethiopia, Nigeria, and Uganda to explore themes related to the existence, exercise, and achievement of family planning choices [22], [23]. Qualitative interviews underscored the ways in which entrenched social norms about fertility shaped women’s contraceptive decisions [23]. Themes arising from qualitative data were used to generate and test a women’s and girls’ sexual and reproductive health empowerment index, including motivations to use contraception [22]. The question assessing perceptions of contraceptive-induced fertility impairment was extracted from the index for this analysis. We dichotomized responses, opposing those who strongly agreed or agreed with the statement from those who strongly disagreed, disagreed, neither agreed nor disagreed, or responded “do not know.” Interviewers selected “do not know” if the respondent was unsure the extent to which they agreed with the statement or were unable to answer, whereas respondents who were ambivalent were categorized as “neither agree nor disagree.”

We explored sociodemographic and reproductive factors related to perceptions of contraceptive-induced fertility impairment. Sociodemographic characteristics included age (15–19, 20–29, 30–39, 40–49), education (none, primary, secondary, higher), marital status (currently married/cohabiting with a man, divorced/widowed, never married), wealth (a tertile based on household assets), and urban/rural residence. Reproductive characteristics included parity (0, 1–2, 3–4, 5 or more) and desired fertility (wants a/another child, wants no more children, undecided/do not know). We considered two measures related to contraceptive practices or intentions, including any current use of medicalized or hormonal contraception (i.e., implants, IUDs, injectables, pills, emergency contraception, henceforth referred to as medicalized contraception), and intention to use any contraception in the next 12 months among nonusers. We focused on medicalized contraception—a term we created to refer to this subset of methods—due to our hypothesis that these hormonal or inserted devices would be most likely to generate concerns about contraceptive-induced fertility impairment, as they interfere with reproductive functions, as opposed to nonhormonal barrier methods or traditional methods that do not. This is also consistent with the literature evaluating fecundity following contraceptive discontinuation, which uses barrier or traditional method users as the reference group given the absence of hormones or devices that might impact time-to-conception [7], [8].

### 2.3 Analysis

This analysis focused on perceived contraceptive-induced fertility impairment among presumably fecund women; thus, we excluded women who reported female sterilization as their contraceptive method, stated they could not become pregnant, self-identified as menopausal, or had a hysterectomy.

We first explored distributions of women’s perceptions of contraceptive-induced fertility impairment across the five Likert scale responses. We then examined bivariable associations using design-based F-tests with our dichotomous version of this variable and conducted multivariable logistic regression to identify correlates of these perceptions. We examined collinearity between covariates and found that no variables’ correlations were greater than 0.7, the threshold commonly used in identifying collinearity. Thus, we retained all covariates in subsequent multivariable analyses.

Next, we explored the association between perceptions of contraceptive-induced fertility impairment and contraceptive behaviors and intentions. To determine the relationship between this belief and current use of medicalized contraception, we restricted the sample of presumably fecund women to those at risk of unintended pregnancy (i.e., sexually active in the last year, nonpregnant, and not wanting a birth in the next 12 months) and conducted bivariable analyses using design-based F-tests and multivariable logistic regression. We then explored bivariable and multivariable associations between perceptions of contraceptive-induced fertility impairment and intentions to use contraception at any time in the future among presumably fecund noncontraceptive users who wanted a/another child. In sensitivity analyses, we reran multivariable logistic regressions after recategorizing women who responded “do not know” to the question on perceptions of contraceptive-induced fertility impairment with those who strongly agreed/agreed given that these respondents were actually *least* likely to be using or intending to use contraception in bivariable analyses. Relatedly, we reran these models using the 5-category Likert scale response options as indicator variables to evaluate the presence of a dose-response relationship. Lastly, among presumably fecund women who believed contraceptives could cause fertility impairment, we estimated the proportion using medicalized contraception and used bivariable analyses to compare users to nonusers to identify the characteristics of women who use medicalized contraception in spite of their fertility impairment concerns.

We conducted all analyses separately by site. We used the Taylor linearization method to account for the complex sampling design, adjusting for geographic clustering, and applied survey weights to account for each woman’s probability of selection. We set *p*-values<0.05 as significant a priori. All analyses were conducted in Stata 17.0.

## 3 Results

**Fig. 1:**
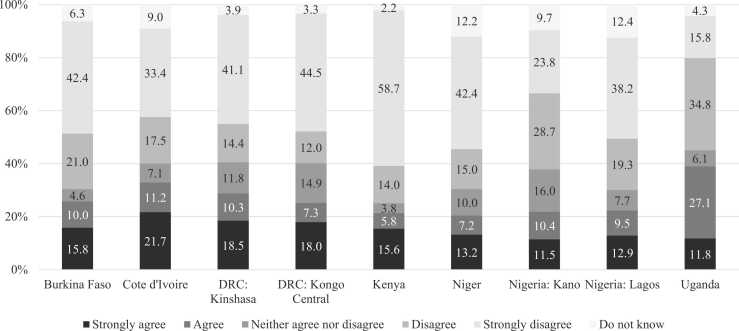
Among presumably fecund women aged 15 to 49 in nine sub-Saharan Africa geographies, the level of agreement with the statement, “If I use family planning, I may have trouble getting pregnant next time I want to,” by site, 2019–2021 (PMA data)^1^. ^1^Burkina Faso n = 6354; Cote d′Ivoire n = 3943; DRC, Kinshasa n = 2516; DRC, Kongo Central n = 1700; Kenya n = 8962; Niger n = 3488; Kano, Nigeria n = 1062; Lagos, Nigeria n = 1435; Uganda n = 3774; PMA = Performance Monitoring for Action.

Between 20% and 30% of women agreed or strongly agreed that contraception would lead to later difficulties becoming pregnant in seven study sites, rising to 33.0% in Cote d′Ivoire and 39.0% in Uganda, while 15.8% in Uganda to 58.7% in Kenya *strongly disagreed* with that statement (Fig. 1).

We present the characteristics of presumably fecund reproductive-aged women by site in Table 1. We observed wide variation across sites in the proportion of women currently using medicalized contraception, from 8.2% in Kano to 38.2% in Kenya. Among those not currently using any contraception, more than 55% intended to use contraception in the future in seven sites, although the proportion fell to 28.1% in Niger and 32.3% in Kano.

**Table 1:** Characteristics of presumably fecund women aged 15 to 49 in nine sub-Saharan Africa geographies and the percent who agree with the statement, "If I use family planning, I may have trouble getting pregnant next time I want to," by background characteristics and site, 2019–2021 (PMA data)^a^

		Burkina Faso		Cote d′Ivoire		DRC: Kinshasa		DRC: Kongo Central		Kenya		Niger		Nigeria: Kano		Nigeria: Lagos		Uganda	
		%	% agree	%	% agree	%	% agree	%	% agree	%	% agree	%	% agree	%	% agree	%	% agree	%	% agree
N		6354		3960		2522		1700		8962		3488		1062		1435		3774	
Age																			
	15–19	22.3	**30.7**	21.8	32.1	23.0	31.3	23.4	22.2	22.6	**28.8**	20.1	**15.0**	25.4	16.6	14.2	**17.8**	25.5	41.7
	20–29	33.3	**26.5**	35.2	32.1	38.6	29.2	34.4	24.6	35.4	**19.8**	41.3	**21.5**	35.6	22.8	30.0	**29.8**	38.2	39.0
	30–39	29.1	**23.0**	29.1	34.7	23.4	25.2	29.2	29.6	27.6	**18.2**	26.3	**21.5**	26.0	23.2	36.0	**21.0**	24.7	38.1
	40–49	15.4	**22.5**	13.9	33.1	15.1	29.3	13.0	23.0	14.4	**19.6**	12.3	**23.3**	13.0	26.5	19.7	**16.8**	11.6	34.6
Education																			
	Never	59.0	**23.7**	42.0	34.5	0.4	7.6	10.8	26.5	4.4	**28.1**	68.7	20.9	47.3	*25.3*	2.4	24.9	6.6	41.3
	Primary	18.0	**29.1**	24.3	34.7	7.2	27.3	27.6	28.5	46.4	**22.1**	15.5	18.0	17.5	*20.3*	9.2	21.8	54.3	37.8
	Secondary	21.3	**28.2**	27.8	31.1	72.7	30.1	58.2	23.2	36.4	**21.7**	13.5	21.4	30.2	*16.6*	51.2	23.3	30.8	41.2
	Higher	1.7	**32.0**	5.9	23.7	19.7	24.7	3.3	31.5	12.9	**15.7**	2.2	18.6	5.0	*25.6*	37.2	21.2	8.3	36.3
Marital status																			
	Currently married/cohabiting	75.5	**23.7**	62.2	34.0	41.6	**25.2**	62.1	25.4	58.3	**18.6**	83.8	**21.8**	76.3	22.7	60.7	21.9	58.7	*36.2*
	Divorced or separated/widowed	3.1	**31.8**	4.5	27.4	6.4	**28.3**	9.7	30.8	8.4	**20.0**	3.5	**17.0**	3.7	25.4	5.9	28.2	12.9	*39.5*
	Never married	21.4	**32.2**	33.4	31.8	52.0	**31.7**	28.2	23.1	33.3	**26.7**	12.7	**11.9**	20.1	17.8	33.5	22.2	28.5	*44.5*
Wealth tertile																			
	Poorest	34.0	24.0	28.3	*37.0*	30.6	27.5	41.6	23.7	35.4	*23.2*	31.3	22.2	35.8	*27.8*	34.1	24.4	30.9	38.4
	Middle wealthiest	32.6	24.8	32.5	*33.1*	33.6	32.7	32.5	24.5	34.3	*21.0*	32.2	18.7	39.3	*22.0*	31.8	22.8	33.3	39.6
	Wealthiest	33.4	28.6	39.2	*30.0*	35.8	26.2	25.8	29.0	30.3	*19.7*	36.5	20.5	24.9	*13.1*	34.1	19.9	35.8	38.8
Parity																			
	0	25.2	**34.4**	28.7	34.6	43.3	31.1	25.9	23.0	29.4	**29.4**	21.5	*15.2*	27.7	19.2	35.6	*23.6*	30.3	**45.3**
	1–2	25.5	**24.9**	33.5	34.4	28.6	28.0	31.9	29.4	31.7	**17.8**	23.5	*22.0*	19.2	22.6	29.9	*25.3*	27.4	**38.2**
	3–4	21.7	**25.1**	20.0	32.6	18.5	23.6	22.0	25.4	22.7	**17.0**	23.8	*22.3*	16.2	27.6	27.4	*19.5*	19.4	**34.2**
	5+	27.6	**19.2**	17.8	28.3	9.6	30.3	20.2	22.0	16.1	**19.9**	31.1	*21.4*	36.9	20.8	7.1	*14.4*	22.9	**35.7**
Residence																			
	Rural	77.0	**23.7**	38.9	34.8	–	–	–	–	69.3	21.9	81.2	20.8	63.7	*26.2*	–	–	71.0	39.4
	Urban	23.0	**32.9**	61.1	31.8	–	–	–	–	30.7	20.1	18.8	18.7	36.3	*14.2*	–	–	29.0	37.9
Desire for a/another child																			
	Undecided/do not know	3.5	**19.3**	4.9	*37.8*	3.7	21.8	12.6	*29.9*	3.6	**13.6**	5.3	**7.7**	16.5	24.2	7.2	**17.3**	3.8	**39.0**
	Have a/another child	80.2	**27.3**	80.8	*33.7*	80.3	29.7	63.1	*26.7*	61.7	**24.3**	90.4	**21.2**	69.3	22.0	64.4	**26.6**	72.4	**41.3**
	No more	16.3	**19.7**	14.3	*27.4*	16.0	26.0	24.2	*19.1*	34.7	**17.0**	4.3	**19.1**	14.2	18.6	28.4	**14.0**	23.8	**31.9**
Currently using medicalized contraception^b^																			
	No	77.4	**29.0**	82.2	**36.3**	84.7	29.1	83.9	25.8	61.8	**25.9**	89.4	20.6	91.8	**23.6**	84.6	**24.1**	75.8	**41.9**
	Yes	22.6	**14.8**	17.8	**17.6**	15.3	26.8	16.1	22.4	38.2	**14.0**	10.6	18.9	8.2	**2.8**	15.4	**12.8**	24.2	**29.9**
Intend to use contraception (among nonusers)																			
	No	36.1	**39.3**	42.9	**50.2**	34.5	31.9	41.8	29.2	39.0	**33.0**	71.9	*24.2*	67.7	**31.6**	40.0	**33.7**	27.8	*50.3*
	Yes	63.9	**24.7**	57.1	**28.6**	65.5	30.3	58.2	25.3	61.0	**24.2**	28.1	*18.6*	32.3	**18.1**	60.0	**19.4**	72.2	*41.5*
Total		100.0	25.8	100.0	33.0	100.0	28.8	100.0	25.3	100.0	21.4	100.0	20.4	100.0	21.8	100.0	22.4	100.0	39.0

a Bolding indicates statistically significantly different at the *p* < 0.05 level from design-based F-test, italics indicate significant at *p* < 0.10 level; PMA = Performance Monitoring for Action.

b Medicalized contraception includes implants, IUDs, injectables, pills, and emergency contraception.

Perceptions of contraceptive-induced fertility impairment varied according to women’s reproductive life course and their social background (Table 1 and Appendix Table A1). Younger women were often more likely to believe contraception could cause fertility impairment in bivariable analysis (Table 1). However, multivariable results showed the opposite association as the odds of believing in contraceptive-induced fertility impairment was typically higher for older women, though this relationship was only significant in Cote d′Ivoire and Kano (Appendix Table A1). Higher educational attainment was associated with lower odds of agreement that contraception causes fertility impairment in multivariable results, though only raising to the level of significance in Cote d′Ivoire and Kenya. Bivariable and multivariable results from multiple countries suggested that nulliparous women were more likely to believe in contraceptive-induced fertility impairment than parous women, as well as women who wanted a/another child compared to those who wanted no more children. Those who were not using medicalized contraception were more likely to hold this belief according to both bivariable and multivariable results. Contraceptive nonusers who did not intend to use contraception in the future were more likely to hold perceptions of contraceptive-induced fertility impairment in five sites based on bivariable analyses.

Perceptions of contraceptive-induced fertility impairment were significantly related to contraceptive use and intentions in bivariable and multivariable analyses (Table 2, Table 3). In all geographies, bivariable results indicated current use of medicalized contraception among women at risk of unintended pregnancy was higher for those who did *not* agree that contraceptive use could cause fertility impairment, with significant differences observed in six sites (Table 2). In multivariable analyses, women at risk of an unintended pregnancy who believed contraception could cause fertility impairment had reduced odds of using medicalized contraception compared to those who did not express that belief in five sites; aORs ranged from 0.07 (95% CI 0.01–0.60) in Kano to 0.62 (95% CI 0.51–0.75) in Kenya (Table 3). Among contraceptive nonusers who wanted a/another child, we similarly observed consistently higher percentages of women’s intention to use contraception in the future among those who did not agree that contraceptives could cause fertility impairment compared to those who agreed in bivariable analyses, with six geographies’ differences raising to statistical significance. Multivariable results were consistent, with significant aORs in seven sites that ranged from 0.34 (95% CI 0.19–0.62) in Lagos to 0.66 (95% CI 0.44–0.98) in Niger (Table 3). Results from sensitivity analyses where we included “do not know” responses with those who strongly agreed or agreed were qualitatively similar but aORs for both outcomes were somewhat further from the null in several geographies (estimates not shown). There was also evidence of a dose-response relationship, particularly for current use of medicalized contraception, with stronger perceptions of contraceptive-induced fertility impairment generally associated with lower odds of medicalized contraceptive use or intention to use contraception (estimates not shown).

**Table 2:** Among presumably fecund women aged 15 to 49 in nine sub-Saharan Africa geographies, percent currently using medicalized contraception^a^ and percent intending to use any contraception in the next 12 months by whether agree with the statement, “If I use family planning, I may have trouble getting pregnant next time I want to,” by site, 2019–2021 (PMA data)^b^

		Burkina Faso		Cote d′Ivoire		DRC: Kinshasa		DRC: Kongo Central		Kenya		Niger		Nigeria: Kano		Nigeria: Lagos		Uganda	
		Current use^c^	Intend to use^d^	Current use^c^	Intend to use^d^	Current use^c^	Intend to use^d^	Current use^c^	Intend to use^d^	Current use^c^	Intend to use^d^	Current use^c^	Intend to use^d^	Current use^c^	Intend to use^d^	Current use^c^	Intend to use^d^	Current use^c^	Intend to use^d^
N		3600	3691	2381	2460	1508	1227	1209	763	6067	3592	1581	2816	525	829	801	718	2354	1955
Agree																			
	No	**36.9**	**70.7**	**32.2**	**67.2**	24.8	68.5	24.2	*64.3*	**58.4**	**72.4**	18.6	**31.0**	**20.3**	**37.8**	**29.6**	**73.4**	**40.9**	80.1
	Yes	**20.9**	**53.5**	**15.9**	**42.7**	24.1	66.0	20.3	*53.6*	**43.3**	**58.1**	21.0	**23.3**	**1.5**	**21.1**	**14.2**	**49.5**	**31.3**	71.3
Total		22.6	33.4	65.3	27.1	57.5	24.6	67.7	23.1	61.0	55.7	68.1	18.7	29.3	16.3	33.1	26.4	66.9	37.5

a Medicalized contraception includes implants, IUDs, injectables, pills, and emergency contraception.

b Bolding indicates statistically significantly different at the *p* < 0.05 level from design-based F-test, italics indicate significant at *p* < 0.10 level; PMA = Performance Monitoring for Action.

c Denominator restricted to those at risk of unintended pregnancy (i.e., sexually active in the last year, nonpregnant, and not wanting a birth in the next 12 months).

d Denominator restricted to those not currently using contraception who wanted a/another child.

**Table 3:** Among presumably fecund women aged 15 to 49 in nine sub-Saharan Africa geographies, adjusted odds ratios (aORs) of currently using medicalized contraception^a^ or intending to use any contraception in the next 12 months by whether agree with the statement, “If I use family planning, I may have trouble getting pregnant next time I want to,” by site, 2019–2021 (PMA data)^b^,^c^

		Burkina Faso	Cote d′Ivoire	DRC: Kinshasa	DRC: Kongo Central	Kenya	Niger	Nigeria: Kano	Nigeria: Lagos	Uganda
		aOR (95% CI)	aOR (95% CI)	aOR (95% CI)	aOR (95% CI)	aOR (95% CI)	aOR (95% CI)	aOR (95% CI)	aOR (95% CI)	aOR (95% CI)
Outcome: current contraceptive use^d^		n = 3076	n = 2113	n = 1376	n = 1135	n = 5605	n = 1300	n = 454	n = 714	n = 2122
Agree family planning will cause difficulties getting pregnancy when want (ref. No)		**0.47** **(0.32–0.67)**	**0.43** **(0.30–0.61)**	1.01 (0.68–1.50)	0.78 (0.47–1.29)	**0.62** **(0.51–0.75)**	0.80 (0.51–1.25)	**0.07** **(0.01–0.60)**	**0.47** **(0.30–0.74)**	0.69 (0.47–1.02)
Outcome: intention to use contraception^e^		n = 2976	n = 1898	n = 1021	n = 591	n = 3176	n = 1953	n = 600	n = 523	n = 1576
Agree family planning will cause difficulties getting pregnancy when want (ref. No)		**0.45** **(0.33–0.61)**	**0.40** **(0.29–0.54)**	0.98 (0.68–1.42)	**0.58** **(0.35–0.98)**	**0.53** **(0.42–0.67)**	**0.66** **(0.44–0.98)**	**0.49** **(0.34–0.71)**	**0.34** **(0.19–0.62)**	0.65 (0.35–1.22)

a Medicalized contraception includes implants, IUDs, injectables, pills, and emergency contraception.

b Bold indicates p-value < 0.05, italics indicates p < 0.10; PMA = Performance Monitoring for Action.

c Adjusted for age, education, marital status, wealth, parity, and residence (except in Kinshasa, Kongo Central, and Lagos), as well as fertility desires for contraceptive use analysis.

d Denominator restricted to those at risk of unintended pregnancy (i.e., sexually active in the last year, nonpregnant, and not wanting a birth in the next 12 months).

e Denominator restricted to those not currently using contraception who wanted a/another child.

Despite this strong relationship between perceived contraceptive-induced fertility impairment and contraceptive nonuse, many women endorsing this statement *did* use medicalized contraception. Approximately 20% to 40% of women who had concerns about contraceptive-induced fertility impairment were using medicalized contraception, with greater use in Kinshasa (41.9%) and less use in Niger (11.3%) and Kano (1.8%) (estimates not shown). Compared to those who held this perception but did not use medicalized contraception, current users tended to be older, more educated, currently married, wealthier, have more children, reside in urban areas, and want no more children, though these relationships were often not statistically significant as sample sizes were small (Appendix Table A2).

## 4 Discussion

Our multicountry study findings indicate women’s perception of contraceptive-induced fertility impairment is common across diverse SSA settings, likely acting as a deterrent to using medicalized contraceptive methods. At least one in five women endorsed this belief across the nine geographies studied, rising to one-third in Cote d′Ivoire and nearly 40% in Uganda. Findings indicate this perception varies across the reproductive life course, declining when women no longer want any children. We also find this belief to be correlated with contraceptive practices (specifically use of medicalized contraceptives), even after adjusting for multiple confounders. Taken together, our results expand on existing literature by quantifying the negative correlation between concerns of contraceptive-induced fertility impairment and contraceptive behaviors and intentions at the population level. These relationships had previously only been observed in qualitative or narrowly focused quantitative research [2], [3], [5].

These nationally and regionally representative results underscore the importance of efforts to address beliefs about contraception, while also recognizing women’s legitimate concerns about potential delayed conception following use of certain methods. The repercussions of these beliefs on contraceptive behaviors are perhaps not surprising in contexts where pressure to bear children is significant and where infertility is common and often goes untreated given a dearth of services [13], [24], [25], [26]. Approaches to fully support women in achieving their reproductive goals need to expand beyond pregnancy prevention and consider women’s fertility concerns, accounting for the local representations of health, fertility, and pregnancy that inform contraceptive decisions [27]. Efforts to reduce unintended pregnancies will be futile without recognizing the critical role that contraceptive beliefs and concerns about future fertility have on women’s reproductive decision-making, as well as expectations regarding time-to-pregnancy that might not align with typical conception times. Improved contraceptive counseling and reproductive health education can dispel myths and address fears, helping women make fully informed contraceptive choices to better manage their fertility. This is of particular importance in settings with a high prevalence of injectable contraceptive use given the more pronounced delays in conception associated with this method [7], [8]. This distinction is critical as evidence suggests beliefs of contraceptive-induced infertility are perpetuated within social networks and therefore amplified in communities where injectables are more prevalent [28]. Future work should examine this phenomenon.

This study quantifies population-level beliefs about contraception’s perceived negative effect on future fertility and their relationship with actual use or intentions to use, across a diversity of contexts with different patterns of childbearing and contraceptive practices. This work also builds upon prior efforts to explore women’s motivations to use or abstain from contraception, especially medicalized contraception, extending our understanding of how these concepts operate among diverse populations [1], [23], [28].

Although we excluded women who were infecund (e.g., had sterilization/hysterectomy or were menopausal), we lacked information on prior fertility-related challenges; thus, we cannot determine the directionality of the association between perceptions of contraceptive-induced fertility impairment and contraceptive practices. Contraceptive use may inform perceptions about contraceptive effects on fertility or vice versa. Future work could longitudinally examine these relationships. For women who did not wish to have any (more) children, they may have had difficulty responding to the question on potential contraceptive-induced fertility impairment in the context of future desired fertility. Relatedly, our measure of pregnancy intentions relied on a simple, direct, timing-based question that lacked validation, despite it being widely used. Additionally, the question about intention to use contraception does not specify the method; thus, these relationships may be stronger if we were able to explore intention to use a medicalized contraceptive method.

Findings from this study can help improve reproductive health programs to address the needs of women and girls more effectively, specifically by addressing concerns about contraception to help them achieve their reproductive goals. Ideally, such efforts should also include infertility-related services to ensure person-centered reproductive health care to address the full extent of women’s reproductive needs.

## Data availability

Data for this study are publicly available at pmadata.org; we relied on each site’s Phase 1 female datasets. Anyone can access these data after completing a brief request form at https://www.pmadata.org/data/available-datasets.

## Figures and Tables

**Table A1:** Among presumably fecund women aged 15 to 49 in nine sub-Saharan Africa geographies, adjusted odds ratios (aORs) of agreeing with the statement, "If I use family planning I may have trouble getting pregnant next time I want to," by site, 2019–2021 (PMA data)^a^

		Burkina Faso	Cote d′Ivoire	DRC: Kinshasa	DRC: Kongo Central	Kenya	Niger	Nigeria: Kano	Nigeria: Lagos	Uganda
		aOR (95% CI)	aOR (95% CI)	aOR (95% CI)	aOR (95% CI)	aOR (95% CI)	aOR (95% CI)	aOR (95% CI)	aOR (95% CI)	aOR (95% CI)
N		6354	3943	2516	1700	8962	3488	1062	1435	3774
Age (ref.15–19)										
	20–29	1.18 (0.89–1.55)	*1.24 (0.96–1.61)*	1.08 (0.84–1.40)	1.10 (0.69–1.77)	1.05 (0.83–1.34)	1.27 (0.85–1.89)	1.79 (0.87–3.66)	**1.80 (1.10–2.94)**	1.44 (0.72–2.86)
	30–39	1.30 (0.92–1.85)	**1.75 (1.23–2.51)**	1.03 (0.73–1.44)	*1.85 (0.91–3.79)*	1.16 (0.86–1.56)	1.32 (0.80–2.17)	*2.89 (0.92–9.11)*	1.31 (0.74–2.31)	1.77 (0.75–4.19)
	40–49	*1.53 (0.96–2.43)*	**1.83 (1.20–2.81)**	1.28 (0.90–1.84)	1.46 (0.66–3.18)	1.19 (0.83–1.72)	*1.67 (0.91–3.04)*	**3.79 (1.30–11.06)**	1.15 (0.55–2.39)	1.64 (0.58–4.63)
Education (ref. Never)										
	Primary	1.15 (0.91–1.46)	0.98 (0.77–1.25)	4.78 (0.49–46.47)	1.04 (0.58–1.85)	0.80 (0.56–1.14)	0.94 (0.63–1.40)	1.00 (0.58–1.73)	1.09 (0.54–2.19)	0.84 (0.52–1.35)
	Secondary	0.86 (0.66–1.11)	0.85 (0.66–1.10)	4.76 (0.53–42.87)	0.68 (0.40–1.15)	**0.63 (0.43–0.92)**	1.44 (0.92–2.27)	1.15 (0.36–3.66)	0.97 (0.45–2.11)	0.86 (0.42–1.74)
	Higher	0.75 (0.51–1.14)	**0.56 (0.34–0.92)**	3.52 (0.38–32.76)	0.90 (0.38–2.14)	**0.45 (0.29–0.68)**	1.20 (0.74–1.94)	2.87 (0.48–17.21)	0.89 (0.43–1.85)	0.64 (0.25–1.62)
Marital status (ref. Currently married/cohabiting)										
	Divorced or separated/widowed	1.29 (0.88–1.89)	**0.70 (0.49–0.98)**	1.16 (0.69–1.96)	1.28 (0.86–1.91)	1.05 (0.83–1.33)	0.73 (0.36–1.50)	0.98 (0.50–1.93)	1.49 (0.87–2.55)	1.15 (0.68–1.96)
	Never married	0.83 (0.60–1.14)	0.91 (0.64–1.30)	*1.38 (1.00–1.91)*	0.91 (0.58–1.43)	0.95 (0.77–1.18)	*0.55 (0.28–1.08)*	1.10 (0.44–2.78)	*0.62 (0.38–1.00)*	1.13 (0.63–2.03)
Wealth tertile (ref. Poorest)										
	Middle wealthiest	1.00 (0.73–1.37)	0.83 (0.61–1.13)	1.29 (0.89–1.87)	1.03 (0.68–1.56)	0.98 (0.83–1.16)	0.84 (0.65–1.07)	0.81 (0.46–1.44)	0.95 (0.63–1.42)	1.10 (0.91–1.33)
	Wealthiest	0.86 (0.55–1.34)	**0.72 (0.52–0.98)**	1.00 (0.69–1.43)	1.39 (0.70–2.77)	0.97 (0.77–1.22)	0.96 (0.58–1.57)	0.47 (0.19–1.18)	0.85 (0.49–1.47)	1.02 (0.64–1.62)
Parity (ref. 0)										
	1–2	**0.55 (0.39–0.79)**	0.80 (0.61–1.05)	0.97 (0.61–1.54)	1.08 (0.69–1.70)	**0.62 (0.49–0.78)**	1.15 (0.67–1.96)	1.00 (0.46–2.18)	0.84 (0.54–1.31)	0.70 (0.34–1.48)
	3–4	**0.55 (0.38–0.80)**	**0.60 (0.42–0.88)**	0.87 (0.58–1.31)	0.75 (0.49–1.14)	**0.59 (0.44–0.80)**	1.15 (0.64–2.06)	1.26 (0.48–3.33)	0.97 (0.52–1.82)	0.57 (0.22–1.47)
	5+	**0.40 (0.26–0.63)**	**0.44 (0.27–0.71)**	1.27 (0.75–2.16)	*0.56 (0.29–1.10)*	**0.65 (0.46–0.91)**	1.05 (0.56–1.91)	0.57 (0.24–1.36)	0.78 (0.35–1.75)	0.63 (0.24–1.60)
Residence (ref. Rural)										
	Urban	**1.71 (1.18–2.47)**	1.02 (0.69–1.51)	–	–	1.01 (0.76–1.32)	0.93 (0.50–1.75)	0.54 (0.18–1.69)	–	0.90 (0.49–1.68)
Desire for a/another child (ref. Undecided/do not know)										
	Have a/another child	**1.72 (1.13–2.62)**	0.73 (0.44–1.23)	1.57 (0.67–3.65)	0.92 (0.54–1.57)	**1.95 (1.24–3.07)**	**2.98 (1.60–5.54)**	1.03 (0.48–2.19)	**1.89 (1.07–3.34)**	1.11 (0.53–2.33)
	No more	1.32 (0.84–2.08)	*0.62 (0.36–1.07)*	1.27 (0.51–3.13)	*0.63 (0.37–1.09)*	1.41 (0.87–2.28)	**2.31 (1.03–5.17)**	0.76 (0.31–1.83)	0.85 (0.45–1.61)	0.75 (0.34–1.65)
Current medicalized contraceptive use (ref. No)										
	Yes	**0.47 (0.34–0.64)**	**0.41 (0.30–0.56)**	0.98 (0.68–1.40)	0.80 (0.49–1.31)	**0.58 (0.49–0.68)**	0.78 (0.53–1.16)	**0.12 (0.02–0.60)**	**0.55 (0.35–0.87)**	*0.66 (0.42–1.06)*

a Bold indicates *p*-value < 0.05, italics indicates *p* < 0.10; PMA = Performance Monitoring for Action.

**Table A2:** Among presumably fecund women aged 15 to 49 at risk of becoming pregnant in nine sub-Saharan Africa geographies who agree with the statement, “If I use family planning, I may have trouble getting pregnant next time I want to,” characteristics of current medicalized contraceptive users and nonusers^a^, by site, 2019–2021 (PMA data)^b^

		Burkina Faso		Cote d′Ivoire		DRC: Kinshasa		DRC: Kongo Central		Kenya		Niger		Nigeria: Kano		Nigeria: Lagos		Uganda	
		User	Nonuser	User	Nonuser	User	Nonuser	User	Nonuser	User	Nonuser	User	Nonuser	User	Nonuser	User	Nonuser	User	Nonuser
N		206	575	93	578	86	277	49	207	420	583	76	195	2	78	21	131	245	510
Age																			
	15–19	*8.2*	*14.7*	*16.5*	*18.9*	11.5	16.0	4.3	19.9	**5.5**	**20.1**	3.1	10.2	0.0	4.5	4.5	6.4	9.1	16.9
	20–29	*35.4*	*41.8*	*25.9*	*41.5*	36.0	48.8	43.6	37.1	**38.9**	**36.6**	51.8	50.1	100.0	46.1	35.0	42.7	44.9	41.0
	30–39	*38.6*	*26.3*	*32.8*	*27.0*	33.5	22.7	43.3	33.8	**40.7**	**22.6**	35.6	23.9	0.0	28.7	48.8	33.3	34.1	26.9
	40–49	*17.8*	*17.2*	*24.8*	*12.6*	18.9	12.6	8.8	9.2	**14.9**	**20.8**	9.5	15.7	0.0	20.7	11.8	17.7	11.9	15.2
Education																			
	Never	**50.6**	**65.3**	39.3	41.5	14.1	6.0	7.0	10.8	**5.2**	**6.9**	64.6	72.0	**0.0**	**67.4**	45.1	4.2	5.9	9.2
	Primary	**29.1**	**14.9**	29.0	30.1	77.4	77.4	31.8	31.1	**60.4**	**46.0**	14.6	15.8	**0.0**	**20.6**	54.9	9.4	62.2	54.0
	Secondary	**19.0**	**17.5**	29.6	23.0	8.5	16.6	59.7	54.7	**24.7**	**34.9**	18.2	11.5	**50.0**	**6.2**	45.6	52.2	27.7	28.7
	Higher	**1.2**	**2.2**	2.1	5.3	18.6	10.3	1.5	3.5	**9.7**	**12.2**	2.7	0.7	**50.0**	**5.8**	51.4	34.2	4.2	8.0
Marital status																			
	Currently married/cohabiting	**86.8**	**82.5**	71.2	64.7	**63.3**	**36.6**	63.3	64.7	**82.8**	**50.0**	100.0	97.3	100.0	92.7	68.3	63.2	**76.6**	**62.8**
	Divorced or separated/widowed	**4.4**	**1.6**	3.6	3.3	**3.0**	**5.0**	9.2	12.3	**8.2**	**10.4**	0.0	2.3	0.0	7.3	15.0	7.4	**16.7**	**13.4**
	Never married	**8.9**	**15.9**	25.3	32.0	**33.6**	**58.4**	27.6	23.0	**9.0**	**39.6**	0.0	0.4	0.0	0.0	16.7	29.4	**6.6**	**23.8**
Wealth tertile																			
	Poorest	31.4	30.5	30.9	34.4	*48.1*	*31.2*	23.1	46.8	35.0	41.3	35.4	32.9	0.0	60.9	23.8	37.9	30.9	33.6
	Middle wealthiest	26.9	37.4	33.1	34.9	*21.5*	*40.4*	38.1	27.9	36.9	34.2	19.0	36.3	0.0	26.4	55.7	35.4	41.0	33.8
	Wealthiest	41.8	32.2	36.1	30.7	*30.4*	*28.4*	38.8	25.3	28.0	24.4	45.6	30.7	100.0	12.7	20.5	26.7	28.2	32.6
Parity																			
	0	**6.0**	**15.8**	17.6	23.1	**16.3**	**40.0**	8.4	18.3	**3.4**	**31.5**	**18.5**	**1.1**	0.0	1.3	22.4	25.5	**5.3**	**19.8**
	1–2	**26.6**	**30.7**	30.0	35.1	**35.8**	**28.9**	50.8	39.2	**37.8**	**26.1**	**50.4**	**36.8**	50.0	20.6	39.5	32.7	**31.7**	**29.0**
	3–4	**29.5**	**27.1**	22.1	22.0	**22.7**	**17.7**	20.4	21.1	**32.7**	**21.9**	**31.2**	**27.3**	0.0	28.8	33.1	31.8	**29.8**	**19.8**
	5+	**37.9**	**26.4**	30.3	19.8	**25.1**	**13.4**	20.4	21.4	**26.1**	**20.4**	**30.4**	**34.8**	50.0	49.4	5.0	9.9	**33.2**	**31.4**
Residence																			
	Rural	**65.5**	**76.3**	46.1	45.0	–	–	–	–	**67.8**	**74.4**	**70.5**	**88.1**	**0.0**	**85.4**	–	–	74.2	74.8
	Urban	**34.5**	**23.7**	53.9	55.0	–	–	–	–	**32.2**	**25.6**	**29.5**	**11.9**	**100.0**	**14.6**	–	–	25.8	25.2
Desire for a/another child																			
	Undecided/do not know	*3.3*	*3.7*	**7.1**	**8.7**	**3.7**	**2.6**	15.5	16.3	**3.1**	**2.3**	2.8	1.3	50.0	22.9	7.8	11.1	3.2	3.6
	Have a/another child	*70.1*	*78.7*	**52.3**	**77.1**	**63.2**	**79.6**	56.3	61.9	**45.7**	**62.5**	93.2	91.0	50.0	54.4	45.9	62.5	63.0	67.5
	No more	*26.6*	*17.6*	**40.5**	**14.2**	**33.1**	**17.8**	28.2	21.8	**51.2**	**35.2**	4.0	7.7	0.0	22.7	46.3	26.4	33.8	28.9
Total		100.0	100.0	100.0	100.0	100.0	100.0	100.0	100.0	100.0	100.0	100.0	100.0	100.0	100.0	100.0	100.0	100.0	100.0

a Medicalized contraception includes implants, IUDs, injectables, pills, and emergency contraception.

b Bolding indicates statistically significantly different at the p < 0.05 level from design-based F-test, italics indicate significant at p < 0.10 level; PMA = Performance Monitoring for Action.

## Appendix

See Table A1 and A2.

## References

[1] Sedlander E., Bingenheimer J.B., Thiongo M., Gichangi P., Rimal R.N., Edberg M. (2018). “They destroy the reproductive system”: exploring the belief that modern contraceptive use causes infertility. Stud Fam Plann.

[2] Williamson L.M., Parkes A., Wight D., Petticrew M., Hart G.J. (2009). Limits to modern contraceptive use among young women in developing countries: a systematic review of qualitative research. Reprod Health.

[3] Daniele M.A., Cleland J., Benova L., Ali M. (2017). Provider and lay perspectives on intra-uterine contraception: a global review. Reprod Health.

[4] Castle S. (2003). Factors influencing young Malians' reluctance to use hormonal contraceptives. Stud Fam Plann.

[5] Boivin J., Carrier J., Zulu J.M., Edwards D. (2020). A rapid scoping review of fear of infertility in Africa. Reprod Health.

[6] Payne J.B., Sundstrom B., DeMaria A.L. (2016). A qualitative study of young women's beliefs about intrauterine devices: fear of infertility. J Midwifery Womens Health.

[7] Yland J.J., Bresnick K.A., Hatch E.E., Wesselink A.K., Mikkelsen E.M., Rothman K.J. (2020). Pregravid contraceptive use and fecundability: prospective cohort study. BMJ.

[8] Gemmill A., Berger B., Bradley S.E., Bell S.O. (2023). The relationship between contraceptive method use and return of fecundity among women attempting pregnancy in low- and middle-income countries. Demography.

[9] Favot I., Ngalula J., Mgalla Z., Klokke A.H., Gumodoka B., Boerma J.T. (1997). HIV infection and sexual behaviour among women with infertility in Tanzania: a hospital-based study. Int J Epidemiol.

[10] Cui W. (2010). Mother or nothing: the agony of infertility. Bull World Health Org.

[11] Lunenfeld B., Van Steirteghem A. (2004). Infertility in the third millennium: implications for the individual, family and society: condensed meeting report from the Bertarelli Foundation's second global conference. Hum Reprod Update.

[12] Dyer S.J., Patel M. (2012). The economic impact of infertility on women in developing countries – a systematic review. Facts Views Vis Obgyn.

[13] Inhorn M.C., Patrizio P. (2015). Infertility around the globe: new thinking on gender, reproductive technologies and global movements in the 21st century. Hum Reprod Update.

[14] Sedlander E., Yilma H., Emaway D., Rimal R.N. (2022). If fear of infertility restricts contraception use, what do we know about this fear? An examination in rural Ethiopia. Reprod Health.

[15] Stevens R., Machiyama K., Mavodza C.V., Doyle A.M. (2023). Misconceptions, misinformation, and misperceptions: a case for removing the “Mis-” when discussing contraceptive beliefs. Stud Fam Plann.

[16] Ganatra B., Gerdts C., Rossier C., Johnson B.R., Tunçalp Ö., Assifi A. (2017). Global, regional, and subregional classification of abortions by safety, 2010–14: estimates from a Bayesian hierarchical model. Lancet.

[17] Bearak J., Popinchalk A., Ganatra B., Moller A.-B., Tunçalp Ö., Beavin C. (2020). Unintended pregnancy and abortion by income, region, and the legal status of abortion: estimates from a comprehensive model for 1990–2019. Lancet Glob Health.

[18] Hellwig F., Coll C.V., Ewerling F., Barros A.J. (2019). Time trends in demand for family planning satisfied: analysis of 73 countries using national health surveys over a 24-year period. J Glob Health.

[19] FP2030 (2022). FP2030's data hub.

[20] The World Bank (2022). https://data.worldbank.org/indicator/SP.DYN.TFRT.IN.

[21] Zimmerman L., Olson H., Tsui A., Radloff S. (2017). PMA2020: rapid turn‐around survey data to monitor family planning service and practice in ten countries. Stud Fam Plann.

[22] Moreau C., Karp C., Wood S.N., Galadanci H., Kibira S.P.S., Makumbi F. (2020). Reconceptualizing women's and girls' empowerment: a cross-cultural index for measuring progress toward improved sexual and reproductive health. Int Perspect Sex Reprod Health.

[23] Karp C., Wood S.N., Galadanci H., Kibira S.P.S., Makumbi F., Omoluabi E. (2020). ‘I am the master key that opens and locks’: presentation and application of a conceptual framework for women’s and girls’ empowerment in reproductive health. SSM.

[24] Mascarenhas M.N., Flaxman S.R., Boerma T., Vanderpoel S., Stevens G.A. (2012). National, regional, and global trends in infertility prevalence since 1990: a systematic analysis of 277 health surveys. PLoS Med.

[25] Cox C., Thoma M., Tchangalova N., Mburu G., Bornstein M., Johnson C. (2022). Infertility prevalence and the methods of estimation from 1990 to 2021: a systematic review and meta-analysis. Hum Reprod Open.

[26] Rutstein S.O., Shah I.H. (2004). Infecundity, infertility, and childlessness in developing countries: ORC Macro, MEASURE DHS.

[27] Schwarz J., Dumbaugh M., Bapolisi W., Ndorere M.S., Mwamini M.-C., Bisimwa G. (2019). “So that's why I'm scared of these methods”: Locating contraceptive side effects in embodied life circumstances in Burundi and eastern Democratic Republic of the Congo. SSM.

[28] Sedlander E., Bingenheimer J.B., Lahiri S., Thiongo M., Gichangi P., Munar W. (2021). Does the belief that contraceptive use causes infertility actually affect use? Findings from a social network study in Kenya. Stud Fam Plann.

